# Supplementary material for: Convergent evolution of oxidized sugar metabolism in commensal and pathogenic microbes in the inflamed gut
Source: Nat Commun. 2025 Jan 28;16:1121. doi: 10.1038/s41467-025-56332-9 (PMC11775122; doi:10.1038/s41467-025-56332-9)
Supplement: Supplementary file 1 — Supplementary Information [file 41467_2025_56332_MOESM1_ESM.pdf]

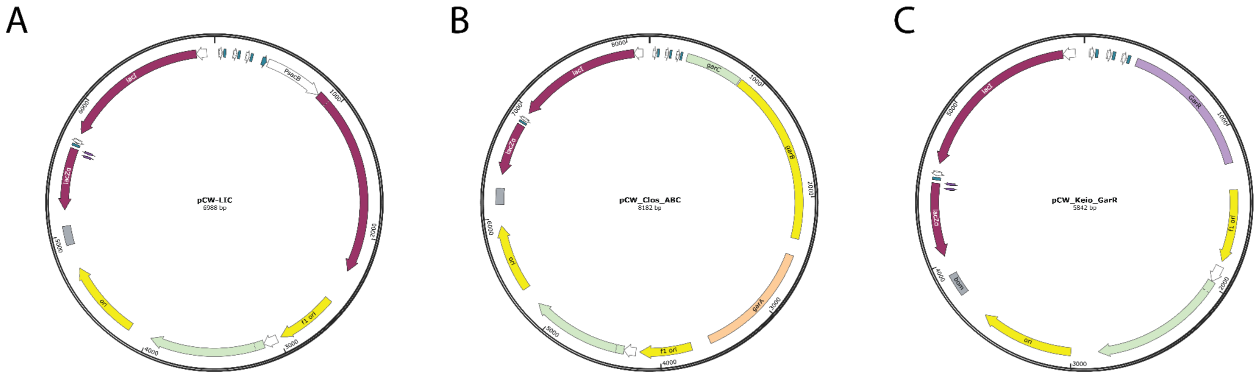


**Supplementary Figure 1: Plasmid maps of constructs.** A) pCW-lic vector backbone.[31] B) pCW_Clos_ABC vector containing the ATP binding cassette genes *garA, garB*, and *garC* from *E. clostridioformis* ligated into the pCW-lic vector backbone via Gibson assembly. C) pCW_Keio_GarR vector containing the tartronate semialdehyde reductase *garR* gene from *E. coli* ligated into the pCW-lic backbone. Pale green arrows represent an ampicillin resistance gene. Maps created using SnapGene.[49]

A

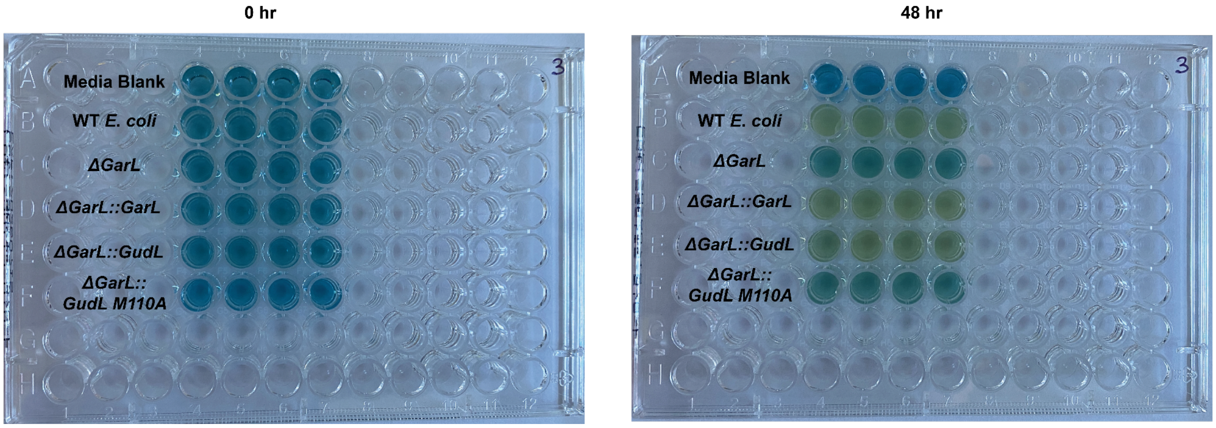


**Supplementary Figure 2: Full images of 96-well plates shown in Figure 3A.** Fermentation media with 10 g/L galactarate inoculated with *E. coli* BL21, *ΔgarL, ΔgarL::garL, ΔgarL::Clos-gudL* or *ΔgarL::gudL-M110A* at 25℃ for 48 hours

A

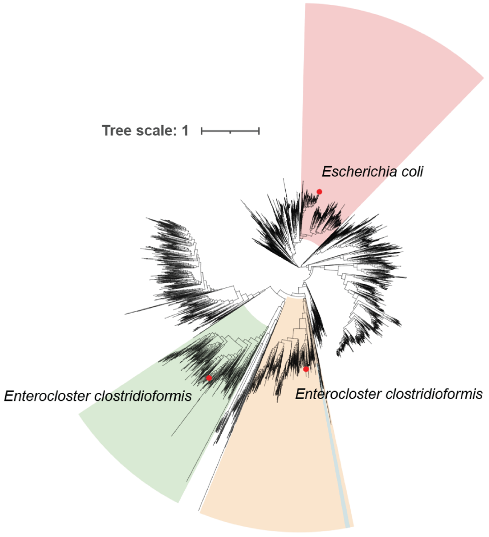


**Supplementary Figure 3: Gene tree of GarK demonstrating polyphyly.** The tree was constructed using IQ-TREE2 with 1,000 bootstrap replicates, incorporating the top 5,000 homologs of *E. coli* and all copies of *E. clostridioformis* GarK. Each putative clade is highlighted in a unique color.

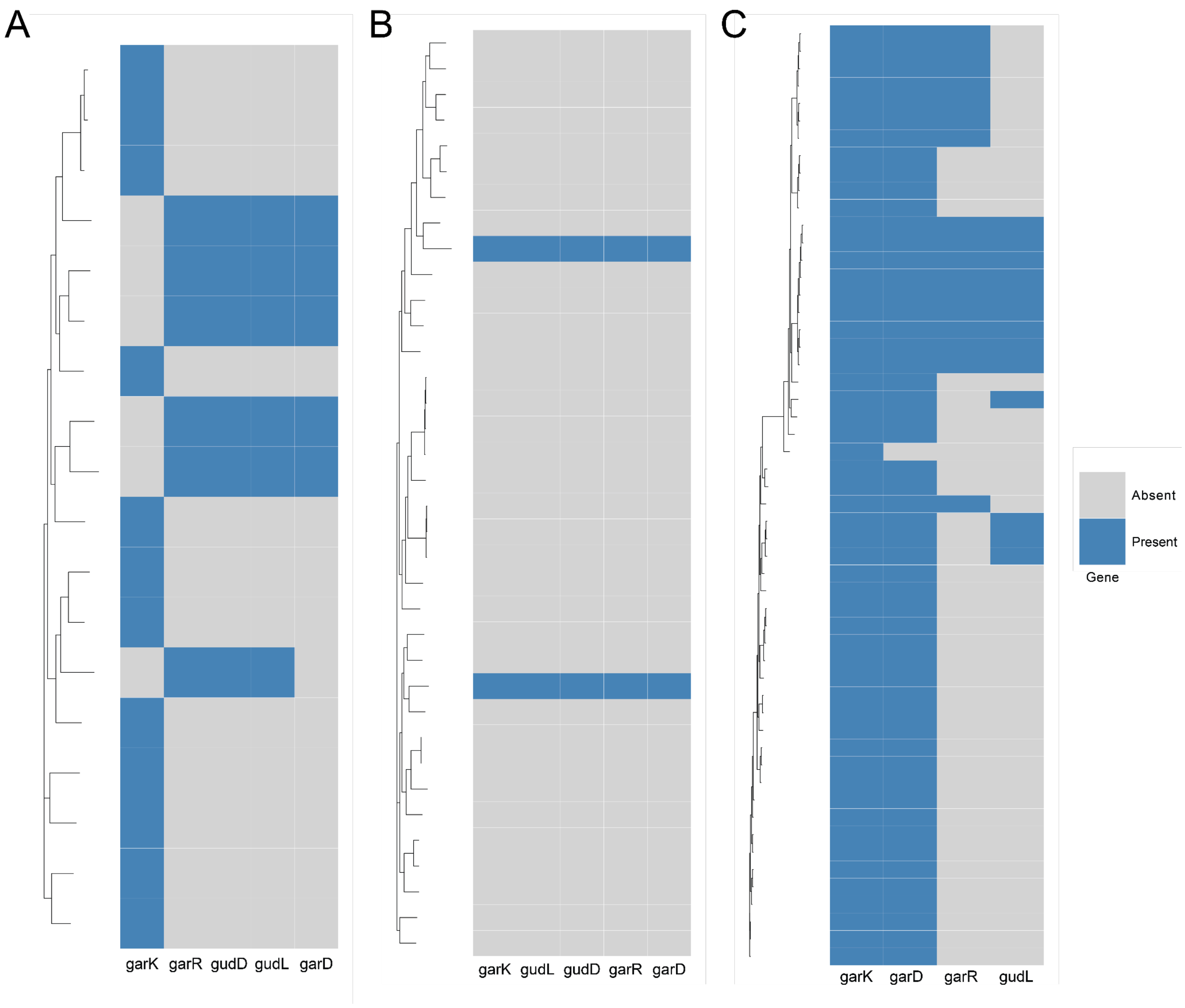


**Supplementary Figure 4: Heatmap showing presence-absence of *gud/gar* genes.** A) *Fusobacterium mortiferum*, B) *Fusobacterium polymorphum* and C) *Enterocloster bolteae* with phylogenies showing each strain, generated by Roary (v3.11.2).

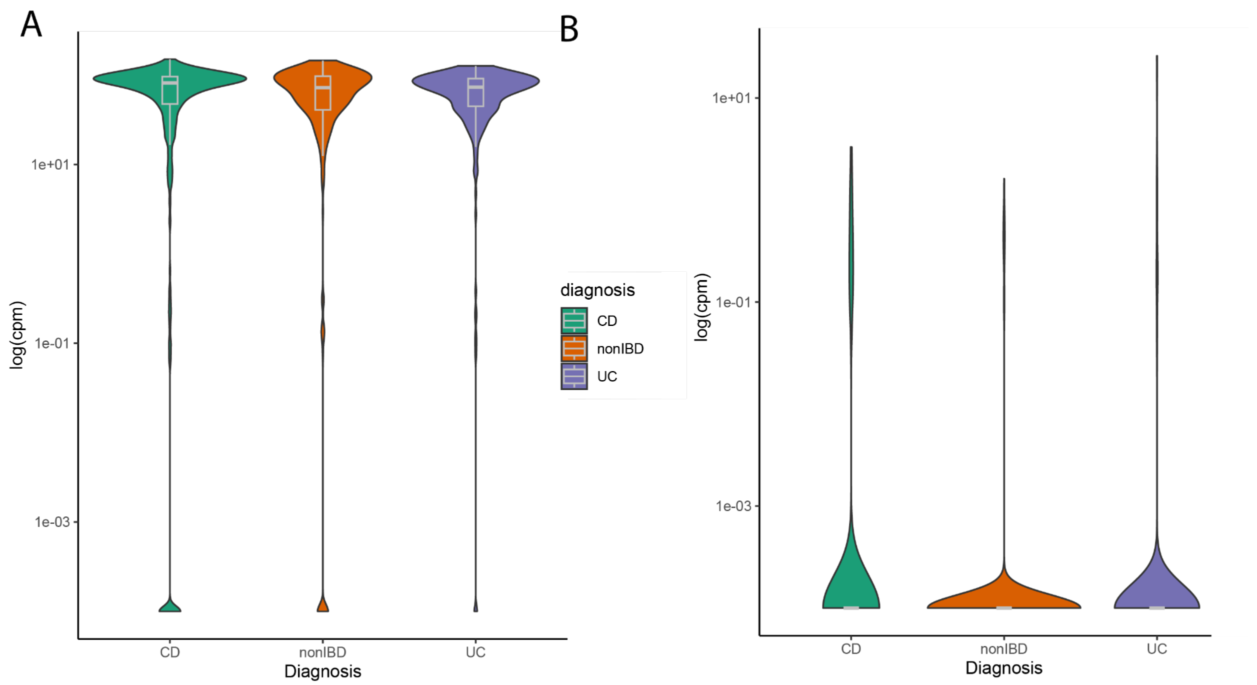


**Supplementary Figure 5: Metagenomic and metatranscriptomic analysis of *garL* in IBD and non-IBD patients.** A) Metagenomic (UC n=337, CD n=565, non-IBD n=359) analyses of *garL* (Mann Whitney U Test, CD *p=8.808e-05 W=115671,* UC *p=0.07631 W=56227,* UC-CD *p=0.03518 W=102641*) and B) Metatranscriptomic analysis of genes *garL* in UC, CD, or non-IBD patients (UC n=166, CD n=270, non-IBD n=570). (Mann Whitney U Test, CD *p=1.752e-08 W=99977,* UC *p=0.2358 W=57046,*UC-CD *p=0.005956 W=28776*).

**Supplementary Table 1: Bacterial strain table**

| Product Identifier | Species | Strain |
|---|---|---|
| HM-317 | *Enterocloster clostridioformis* | WAL-7855 |
| HM-309 | *Clostridium symbiosum* | WAL-14163 |
| HM-315 | *Enterocloster citroniae* | WAL-17108 |
| HM-1057 | *Mediterraneibacter lactaris* | CC59-002D |
| HM-714 | *Bacteroides fragilis* | CL03T12C07 |

**Supplementary Table 2: Primers used in construct development**

| Species | Gene(s) | Direction | Sequence (5’ > 3’) |
|---|---|---|---|
| *E. clostridioformis* WAL7855 | *garABC* | Forward | catcgatgcttaggaggtcaaATGTTTATTAAAAAGTATGGGGATATC |
| | | Reverse | ttgacagcttatcagcgataTTATTTTGCTAAATCGTCTGC |
| | *gudL* | Forward | catcgatgcttaggaggtcaaATGAATACAGATTTTATCAAGGG |
| | | Reverse | ttgacagcttatcagcgataTTAAATCAGCCCTGCCTG |
| | *garR* | Forward | catcgatgcttaggaggtcaaATGAAAGTAGGTTTTATCGG |
| | | Reverse | ttgacagcttatcagcgataTTATGATTCAACTGTAGTATTGG |
| *E. coli* BW25113 | *gudP* | Forward | taagcacatATGCAGGAGTGCAACAATGAGTTCTTTAAGTC |
| | | Reverse | taagcaggtaccGTGTCGCCATCTTATTGCCCC |

| | | | |
|---|---|---|---|
| | *garP* | Forward | catcgatgcttaggaggtcaaATGATTCTGGACACCGTTG |
| | | Reverse | ttgacagcttatcagcgataTTATTTCTGCAATTCCATACG |
| | *garL* | Forward | catcgatgcttaggaggtcaGATGAATAACGATGTTTTCCC |
| | | Reverse | ttgacagcttatcagcgataTTATTTTTTAAAGGTATCAGCC |
| | *garR* | Forward | catcgatgcttaggaggtcaGATGAAAGTTGGTTTTATTGGC |
| | | Reverse | ttgacagcttatcagcgataTTAACGAGTAACTTCGAC |
