# Supplementary material for: Convergent evolution of oxidized sugar metabolism in commensal and pathogenic microbes in the inflamed gut
Source: Nat Commun. 2025 Jan 28;16:1121. doi: 10.1038/s41467-025-56332-9 (PMC11775122; doi:10.1038/s41467-025-56332-9)
Supplement: Supplementary file 2 — Description of Additional Supplementary Files [file 41467_2025_56332_MOESM2_ESM.docx]

**File Name: Supplementary Data 1**

**Description:** Supplementary Data 1 contains three tabs. The Sugar Oxidation Tree tab contains the NCBI accession numbers and taxonomic information for the genomes analyzed. The Metagenomics Metadata tab contains the metadata underlying the metagenomic data, including the diagnoses, total reads, the reads mapped to the *gud/gar* pathway, the reads mapped to *gudL* and the reads mapped to *garL.* The Metatranscriptomics Metadata tab contains the metadata underlying the metatranscriptomics data, including the diagnoses, total reads, the reads mapped to the *gud/gar* pathway, the reads mapped to *gudL* and the reads mapped to *garL.*
